# Dynamic clustering via branched deep learning enhances personalization of stress prediction from mobile sensor data

**DOI:** 10.1038/s41598-024-56674-2

**Published:** 2024-03-19

**Authors:** Yunfei Luo, Iman Deznabi, Abhinav Shaw, Natcha Simsiri, Tauhidur Rahman, Madalina Fiterau

**Affiliations:** 1 Manning College of Information and Computer Science, University of Massachusetts Amherst, 140 Governors Drive, Amherst, MA 01003 USA; 2 Halıcıoğlu Data Science Institute, University of California San Diego, 9500 Gilman Dr, San Diego, CA 92093 USA; 3 Computer Science, Stanford University, 450 Jane Stanford Way, Stanford, CA 94305 USA

**Keywords:** Machine learning, Multitask learning, Stress prediction, Mobile computing, Human behaviour, Stress and resilience, Human behaviour, Stress and resilience

## Abstract

College students experience ever-increasing levels of stress, leading to a wide range of health problems. In this context, monitoring and predicting students’ stress levels is crucial and, fortunately, made possible by the growing support for data collection via mobile devices. However, predicting stress levels from mobile phone data remains a challenging task, and off-the-shelf deep learning models are inapplicable or inefficient due to data irregularity, inter-subject variability, and the “cold start problem”. To overcome these challenges, we developed a platform named Branched CALM-Net that aims to predict students’ stress levels through dynamic clustering in a personalized manner. This is the *first platform that leverages the branching technique in a multitask setting to achieve personalization and continuous adaptation*. Our method achieves state-of-the-art performance in predicting student stress from mobile sensor data collected as part of the Dartmouth StudentLife study, with a ROC AUC 37% higher and a PR AUC surpassing that of the nearest baseline models. In the cold-start online learning setting, Branched CALM-Net outperforms other models, attaining an average F1 score of 87% with just 1 week of training data for a new student, which shows it is reliable and effective at predicting stress levels from mobile data.

## Introduction

Stress is one of the most common contributors to widespread health problems, making its early identification and continuous monitoring essential for the development of effective treatments and interventions^1^. Chronic stress often causes anxiety disorders, which affect almost 30% of adults at some point in their lives^2^. In addition, prior research suggests that overwhelming stress can lead to deadly conditions such as cardiovascular diseases^3,4^, memory and cognition impairment^5^, and suppression of the immune system^6^. Furthermore, stress has been shown to aggravate metabolic dysfunctions, including insulin resistance as well as disruptions in glucose and lipid homeostasis^7^. For college students in particular, stress has a negative effect both academically and socially^1^.

Due to the serious impact of stress on college students, it would be extremely useful if their level of stress could be continuously monitored by the school’s health staff and the students themselves, to facilitate interventions designed to preempt potential health problems. Since it is not feasible for the staff to perform clinical tests for all the students, such as measuring cortisol levels^8^ at regular intervals throughout the day, using data collected continuously via cell phones and having a system to passively predict users’ levels of stress is a cost-efficient and desired alternative. Studies focusing on this approach^9,10^ have shown that machine learning has a tremendous potential applicability^11^ to predict stress level, as well as other mental conditions^10,12–14^ with recent attempts focusing on the use of deep learning methods^11,15–17^. Among deep learning methods, Long Short-Term Memory (LSTM) networks^18^ and attention-based models^19^ have been the top performers in tasks involving various types of time series data^20–26^. However, there are some major challenges specific to the stress prediction task, including intersubject variability, cold start problem, and data irregularity, which make prior methods ill-suited to this task. We will discuss each of the challenges in detail and present how our proposed method is designed to overcome each of them.

An important problem is that most standard machine learning models assume homogeneity in the data, implying that all collected samples originate from the same distribution. However, the way people experience mental conditions and specifically *stress levels vary significantly from individual to individual*^27^. To address this challenge, personalized models have been introduced and explored^17,28–30^. Techniques involving personalized parameters have been used for a wide range of applications, from intelligent sensing and health status monitoring^31^, to next-word prediction on mobile devices^32^. For the prediction of mental state, recent work such as^17^ and^28^ have shown the importance of considering individual differences and constraints in modeling. We adopt *branched deep learning as a new approach to personalization* and experimentally show that our model attains the best performance at predicting student stress levels with limited data.

Despite promising performance, personalized models often suffer from *the “cold-start” problem*^33^. The cold start problem occurs when new subjects are introduced into a predictive model with personalized parameters. Because the model lacks data on the new subject, it cannot immediately provide a reliable prediction for that subject. Ideally, the new subject could be assigned to a group of subjects who share similar patterns and have well-trained parameters to obtain a reasonable prediction immediately. Previous works in this area^11,17,28–30^ neither evaluate the performance of their proposed system in this cold start scenario nor explore to what extent the parameters should be shared between subjects. To achieve information sharing, we will use a technique called Learn-to-Branch, which was introduced in^34,35^ for Computer Vision applications to find the best paths in neural networks for each task so that the information learned by some particular node(s) will only be shared with similar tasks. To the best of our knowledge, *we are the first to apply Learn-to-Branch to time-series data and use it for personalization.*

Another characteristic of time series data for this task is that they are usually *collected irregularly over time with different features collected with different frequencies*^36–39^. There are many different data modalities that are useful in student stress prediction, including audio, GPS, movement, phone usage statistics, and the duration of sleep and academic-dependent data which may include indicators for the exam period, and the deadline for the next assignment. All of these modalities have been shown to be useful^40^ for student stress prediction. Che et al.^39^ and Shukla et al.^22^ provide an overview of the approaches on modeling data with irregularity using deep learning methods. Here, we adopt a feature engineering method to *regularize the sample rate* followed by dimensionality reduction^41^. This technique is also employed to *mitigate noise in the data* used to train the classification model for predicting student stress levels.

To assess the efficacy of our models and position them within the context of existing research, we used the StudentLife dataset^40^ as our primary data source. Our selection of the StudentLife dataset stemmed from its distinct advantage: an extensive data collection period of approximately 3 months. This prolonged duration stands out in comparison to most depression monitoring studies, which typically gather data over only 1–2 weeks, as acknowledged in^42^. This extended timeframe allowed us to encompass data from students at various stages, including periods characterized by diverse course assignments and examination schedules.

Moreover, the dataset offered the opportunity to incorporate multimodal features into our modeling process. These encompassed physical activity, phone usage, sleep statistics, conversation records, and more. This marked a departure from previous studies that primarily focused on single features, such as location or sociability. By harnessing multiple features simultaneously to train our models, we demonstrated the superior performance of our multimodal learning approach compared to earlier state-of-the-art work, as exemplified by^11^, which primarily concentrated on location-based features.

In the literature on the StudentLife dataset, several significant contributions stand out as valuable benchmarks for comparison. Gatis et al.^11^ introduced an approach named Multilayer Perceptron based on location features (Location MLP), leveraging novel location-based features extracted from the StudentLife dataset, to make stress level predictions. Similarly, Adler et al.^43^ employed Gradient Boosted Decision Trees (GBDT) augmented with statistical features to predict stress levels within the same dataset.

Additionally, the work by^44^ unveiled the PSP-IGR model, which combined a user-specific information-enriched multi-layered perception (MLP) for low-frequency sensor data with a CNN-LSTM model tailored for high-frequency sensor data. Alongside,^45^ demonstrated the utility of XGBoost in stress prediction, enhancing feature potency through the application of Synthetic Minority Oversampling Technique (SMOTE) (Despite exerting diligent efforts, the source codes for the PSP-IGR model^44^ and the work by^45^ eluded our reach. Despite direct communication with the authors and exhaustive online searches, the unavailability of these codes prevented us from conducting a holistic comparison between our results and these two approaches.)

The method in^43^ achieved state-of-the-art performance for binary and three-level stress prediction using five-fold cross-validation. Nevertheless, this work does not take inter-subject variability into account, and the evaluation under the cold-start and continuous learning settings is lacking.

In this work, we propose a method based on hierarchical multitask learning for predicting stress from smartphone data. Our approach addresses each of the identified problems through careful method selection and empirical validation. The standard version of our proposed method is called the Cross-personal Activity LSTM Multitask Network (CALM-Net), a system designed specifically to deal with inter-subject variability. CALM-Net learns personalized parameters by considering each subject as a task in a multitask learning structure. With shared and individual parts, CALM-Net can learn both the shared dynamics of the data between all subjects as well as personalized characteristics. We also propose Branched CALM-Net, a variant of CALM-Net designed to find and share parts of the network among similar subjects. Branched CALM-Net combines multitask learning with the Learn-to-Branch technique to efficiently model both personal and population-level characteristics. Similarly, we develop CATrans-Net and Branched CATrans-Net which use a Transformer model^46^ instead of the LSTM model in the architecture.

We show that Branched CALM-Net achieves *state-of-the-art performance in student stress prediction from as little as 1 week of training data* for an individual subject. We also show the stability and robustness of our system under cold start scenarios and online learning settings when a subject starts with no data and more data are added over time. Specifically, Branched CALM-Net achieves an F1 score of around 0.67 for 3-class stress prediction for a new subject who has only the first week of training data available. With more training data included for a subject, Branched CALM-Net remains the top performer among the tested methods. Thus, Branched CALM-Net enables the accurate detection of high levels of stress from passively collected smartphone data. This detection is a key prerequisite for enabling the deployment of early intervention systems to avoid the development of serious health problems and mitigate the influence of high stress on students’ academic performance.

**Figure 1:**
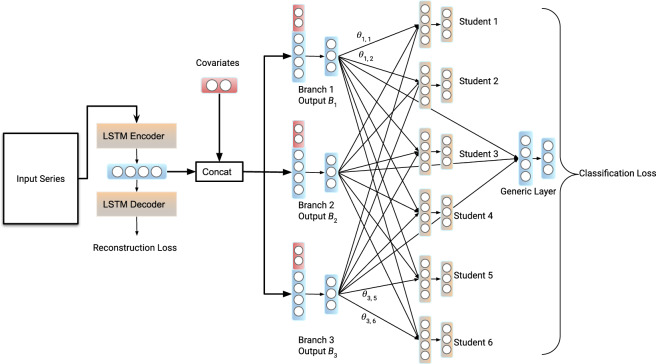
An overview of the structure of CALM-Net enhanced with Learn-to-Branch technique, forming the Branched CALM-Net model. The network consists of an LSTM autoencoder, followed by branches, and multi-layer outputs. The structure includes a generic layer set which models information for all subjects, and separate layers for each subject, obtained by adaptively combining information from multiple branches.

**Figure 2:**
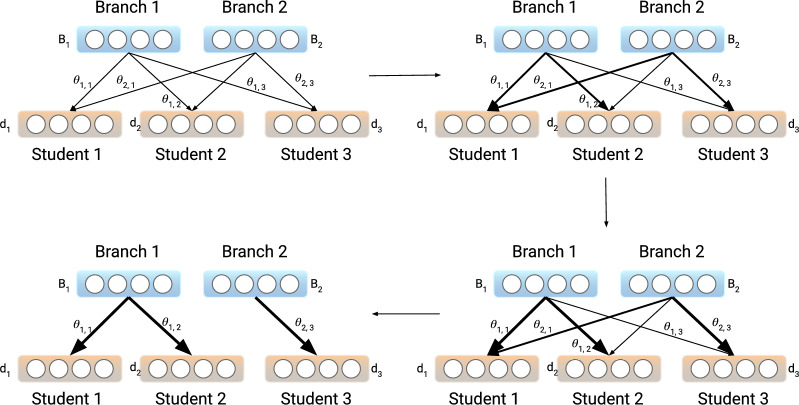
Pipeline of Learning to Branch. The probabilistic weights are uniformly initialized, and the sampling strategy follows the Gumbel-Softmax technique. The branching weights will finally converge to one-hot vectors by using Gumbel-Softmax trick. This branching mechanism can control the information shared between the layers for each student.

## Results

### Experimental setup

The StudentLife data set provides a benchmark for research on stress monitoring. Data have been collected from college students at Dartmouth College, over 9 weeks, containing psychological surveys that include stress level queries, GPS locations, phone usage statistics, audio, and physical activity inference, which are the indicators representing whether the motion is slight or intense based on the raw signal. Table 8 shows the features we choose along with their pre-processing methods. This selection strategy was implemented to create a balanced dataset, thereby minimizing data skewness. By doing so, we aimed to align the total number of samples with the figures reported in the Location MLP baseline study, ensuring consistency and reliability in our data analysis. We observed that many students had less than 35 days with self-reported stress level, which could potentially result in an under-representation of their stress patterns. Simultaneously, one student had more than 200 records of self-reported stress level with which is significantly more data than the others. To avoid data skewness and ensure a balanced analysis, we selected students with a data range that was neither too short nor excessively long –more than 35 days and less than 150 days with self-reported stress records.

There are five stress labels in this data set: (1) no stress at all, (2) feeling well, (3) a little stressed, (4) stressed, (5) very stressed. Due to the high imbalance of the number of labels, we follow^11^ to combine the first two labels as (1) *below median* or *no stress*, the third label as (2) *median stress*, and the last two labels as (3) *above median*.

We evaluate the baselines and our model, CALM-Net (Fig. 1) with Learn-to-Branch (Fig. 2), in two scenarios. First, we consider the case when there is sufficient data for all students to train the models. For this evaluation, we performed five-fold cross-validation of the data, for which the entire dataset is split into five subsets stratified by each student and each stress level. The temporal order of the samples is not important here because the prediction is made with data from a single day as input, meaning the prediction process is independent with respect to data from past and future days. Although the evaluation schema is theoretically sound, it does not account for the practical limitation that future data is unavailable in our training datasets. To address this and validate our model in more realistic scenarios, we performed an additional round of cross-validation. This involved segmenting the data chronologically, ensuring that our model’s effectiveness is tested under conditions that closely resemble real-world applications.

The second scenario corresponds to the cold-start problem, where we conduct leave-one-subject-out cross-validation. In this evaluation, we are simulating the situation where we have a dataset for offline modeling which includes data from all but one of the students, then we conduct prediction on the new student, the left out one, for whom we have no data. We also test the methods in the online learning setting, in which data from the new student is progressively added for training. This last evaluation simulates the scenario after the cold start, determining the amount of student monitoring time needed by each method to be able to predict the stress levels for a new student.

We use the Location MLP^11^, one of the previous state-of-the-art models, as a baseline to ascertain the competence of our model for stress prediction in the StudentLife data set. We also compare against GBDT with statistical features, as used in^43^ for EMA prediction on StudentLife data.

In the cold-start-with-online-learning setting we also compare our results against another baseline: a neural network model that uses student clusters as tasks (named Clustered CALM-Net ). To find these clusters, we use the survey scores to group the students, where the scores are computed from students’ responses to psychological surveys: PHQ-9 for depression, Perceived stress scale (PSS) for stress, Longliness scale, Flourish scale, Positive and Negative Affect Schedule (PANAS), and Big Five Personality Traits.

### Evaluation

We use two different evaluation schemes to benchmark the performance of our models against the baselines. First, we use five-fold cross-validation on the time series of each student and report the resulting average F1 score and Area Under the Receiver Operating Characteristics Curve (AUC)^47,48^. Because the stress labels are very imbalanced (with portions of $22.1\%, 43.4\%, 34.5\%$ for *below median, median, above median* respectively), both the F1 and AUC scores are appropriate for evaluation in such settings^47,49^. This approach of splitting each student’s data into five-folds simulates the situation of warm-start when there is relatively sufficient data available to train personalized models for all the subjects.

We present evaluation results not only on the 3 classes stress prediction which test the models’ ability of differentiating levels of stress, but also on binary detection that test the performance of models on identifying positive (combining labels of *median* and *above median*) and negative cases (*below median*). We validate our models under these two settings to demonstrate that our methods can meet a range of real-life requirements. Conducting binary stress detection is a basic requirement for the application. However, if the school’s mental health staff or students themselves are interested in having different treatments for moderate and high levels of stress, a more fine-grained prediction could be provided with the three-class setting. In this scenario, our approach could provide a more detailed assessment of stress levels, allowing for tailored interventions and support for those in need.

However, as mentioned, having sufficient data is not always the case. For instance, a new student who joined the system will have no or limited training data. The cold-start scenario is important for personalized models and has not been previously considered in prior research on stress prediction^16,17,28^. To evaluate our models in this scenario, we will use a leave-one-subject-out approach, which means that we use one student as our test data in each iteration of evaluation. We report the F1-score, averaged over all students. Furthermore, to simulate the situation where the model continuously collects data about a subject as the subject uses the mobile app, we will train the model on a growing portion of data from the test student in each iteration and test it on the rest of available data from the test student. In the rest of this section, we will discuss the results achieved by our models and baselines in these scenarios. A summary of the evaluation schemes is shown in Table 1.

**Table 1:** Evaluation schemas.

Scenarios	Binary stress detection	Stress level prediction	Stress level prediction with cold start
Labels	Not stressed, stressed	Below median, median, above median	Below median, median, above median
Evaluations	Five-fold cross-validation	Five-fold cross-validation	Leave-one-subject-out validation
Sections	“CALM-Net and branched CALM-Net attain AUC scores of more than 0.8 on student stress detection” section. On binary stress detection, CALM-Net attains precisions higher than $84\%$ when recovering $90\%$ positive cases	“Branched CALM-Net improves upon the SOTA on 3-class stress level prediction” section. Branched CALM-Net improves the state-of–the-art by introducing personalization and dynamic clustering on stress level prediction with 3 classes	“With 1 week of data, branched CALM-Net achieves 17.24% boost in performance over SOTA” section. Branched CALM-Net is the top performer with F1 score of 0.67 when training on 1 week data from new subject

Our evaluation methodologies, along with their descriptions and main takeaways

### CALM-Net and Branched CALM-Net attain AUC scores of more than 0.8 on student stress detection

**Table 2:** Performance evaluation in binary stress detection.

Model	Precision (@Recall$\approx$0.9)	PR AUC	ROC AUC
Location MLP^11^ GBDT^43^ LSTM^18^ Clustered CALM-Net CALM-Net Branched CALM-Net	0.588 ± 0.002 **0.849** ± **0.026** 0.780 ± 0.004 0.817 ± 0.003 **0.843** ± **0.003** **0.845** ± **0.005**	0.674 ± 0.012 0.877 ± 0.031 0.805 ± 0.010 0.877 ± 0.002 **0.933** ± **0.001** **0.931** ± **0.001**	0.580 ± 0.011 0.582 ± 0.064 0.530 ± 0.020 0.684 ± 0.003 **0.807** ± **0.002** **0.805** ± **0.004**
Transformer (Trans)^46,50^ CATrans-Net Branched CATrans-Net	0.780 ± 0.004 **0.851** ± **0.006** **0.851** ± **0.004**	0.786 ± 0.008 **0.932** ± **0.002** **0.933** ± **0.001**	0.501 ± 0.015 **0.805** ± **0.003** **0.805** ± **0.002**

**Figure 3:**
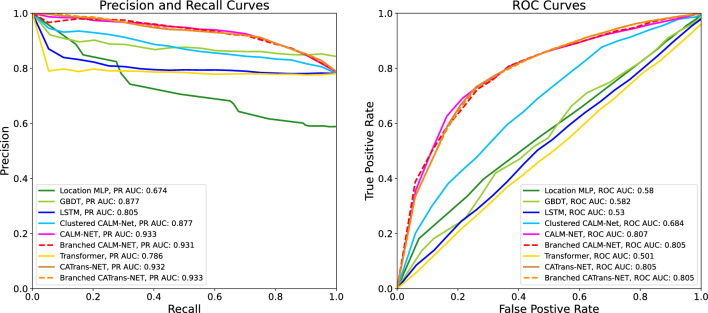
Precision and Recall curves (Left) and ROC curves (Right) of Location MLP, GBDT, LSTM, CALM-Net, and models with Transformer^19^ as backbone (namely CATrans-NET) on the task of binary stress detection.

The labels of *median stress* and *very stressed* are combined. We report the precision of different models for a recall of 0.9 and the Area Under the Curve (AUC) for the precision-recall curve and for the receiver operating characteristic (ROC). The PR and ROC curves are shown in Fig. 3.

[bold] values indicate top performers in each category.

To evaluate our methods and compare them with baselines in the case that there is sufficient data for each student, we present the results achieved by each method in the warm-start setting.

We present the classification performance of our models versus the baselines in this scenario in Table 2. On the left-most column, we present the precision while fixing the recall at $90\%$. Table 2 also shows the performance of Branched CALM-Net, which is the version of our method that uses Learn-to-Branch. In Fig. 3 we show the ROC curve along with the AUC score for Location MLP, GBDT, LSTM, Transformer, CATrans-Net, and CALM-Net models. Empirical results, indicate that our proposed CALM-Net and CATrans-Net significantly outperforms Location MLP, GBDT, Transformer and LSTM, which do not have personalized parameters.

### Branched CALM-Net improves upon the SOTA on 3-class stress level prediction

**Table 3:** The F1 scores for 3-class stress level prediction on the StudentLife dataset using five-fold cross-validation under the warm-start scenario.

Model	F1-score	ROC AUC
Location MLP^11^ GBDT^43^ LSTM CALM-Net	0.388 ± 0.007 0.488 ± 0.033 0.479 ± 0.004 **0.602** ± **0.004**	0.631 ± 0.003 0.603 ± 0.028 0.630 ± 0.002 **0.778** ± **0.002**
Clustered CALM-Net Branched CALM-Net	0.530 ± 0.003 **0.605** ± **0.003**	0.690 ± 0.003 **0.782** ± **0.002**
Transformer (Trans) CATrans-Net Branched CATrans-Net	0.450 ± 0.006 **0.593** ± **0.004** **0.591** ± **0.003**	0.616 ± 0.002 **0.772** ± **0.002** **0.773** ± **0.001**

Performances on the task of stress level prediction with 3 levels (*below median, median, above median*). The top section contains the baseline models with the personalized models. The bottom section is models that have grouping methods introduced. Both F1 and AUC scores are calculated globally using micro-averaging by considering each element of the label indicator matrix as a positive label.

[bold] values indicate top performers in each category.

**Table 4:** The performance on binary class stress level prediction on the StudentLife dataset using chronological five-fold cross-validation.

Model	Precision (@Recall$\approx$0.9)	PR AUC	ROC AUC
LSTM	0.798 ± 0.004	0.832 ± 0.009	0.579 ± 0.013
CALM-Net	**0.835** ± **0.007**	**0.923** ± **0.002**	0.779 ± 0.005
Branched CALM-Net	**0.836** ± **0.006**	**0.925** ± **0.002**	**0.782** ± **0.004**

[bold] values indicate top performers in each category.

**Table 5:** The performance on 3-class stress level prediction on the StudentLife dataset using chronological fivefold cross-validation.

Model	F1-score	ROC AUC
LSTM	0.481 ± 0.006	0.796 ± 0.006
CALM-Net	**0.581** ± **0.006**	**0.858** ± **0.004**
Branched CALM-Net	**0.586** ± **0.005**	**0.863** ± **0.003**

[bold] values indicate top performers in each category.

We additionally provide evaluation results for distinguishing between three stress levels (below median, median, and above median) in Table 3. In this context, we also compare our results to the baseline approach, referred to as Clustered CALM-Net, which explicitly forms student groups. We include this baseline to investigate whether introducing Learning-to-Branch^34^ to dynamically discover implicit groups during the training process can yield performance improvements. According to Table 3, Branched CALM-Net achieves the best performance, which demonstrates the benefit of dynamic clustering.

To further validate our concept and align our evaluation with real-world applications, we conducted an additional round of cross-validation based on chronological data segmentation. This approach ensures that the data is split in a manner that mirrors actual temporal sequences, thereby enhancing the practical relevance of the analysis. The outcomes of this chronological cross-validation are detailed in Tables 4 and 5. Notably, the results corroborate our previous findings in Table 3, demonstrating consistent performance even under more realistic data segmentation scenarios. This consistency reinforces the robustness and applicability of our Branched CALM-Net approach in real-world settings.

### With 1 week of data, Branched CALM-Net achieves 17.24% boost in performance over SOTA

**Figure 4:**
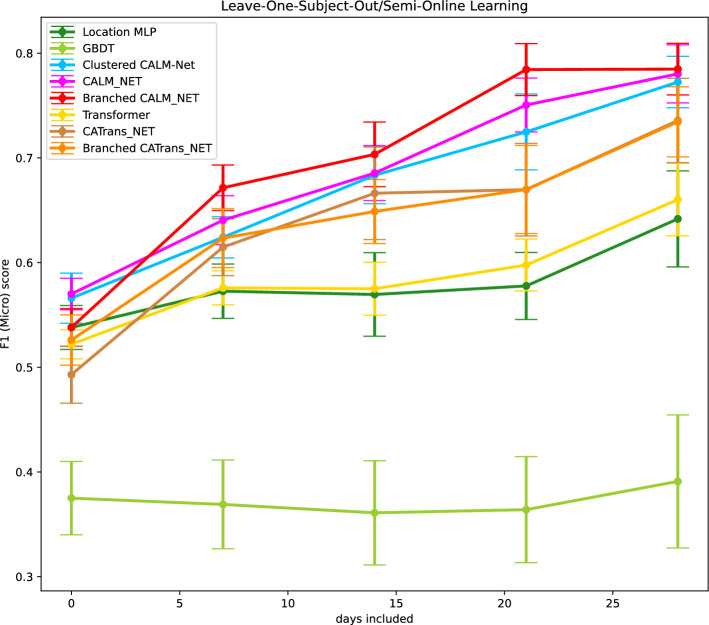
Online learning in the cold-start setting: Performance plotted against the amount of data included in the training set from the left-out student. Error bars indicate the variance of the scores across students. For both CALM-Net and Branched CALM-Net, predictions with zero data included are generated by the generic layer.

To evaluate the performance of the models in the “cold-start” setting, we use leave-one-subject-out cross-validation. In this setting, the data set is split into subsets, referred to as folds, with numbers equal to the number of subjects, and each fold contains the data samples corresponding to one subject or student in this data set. For each fold, we train the model on the data from other students and then validate on the data that belongs to the left-out student. Furthermore, to assess the functionality of our models using continuously collected data from left-out students, we incrementally add the data of each student, 1 week at a time, and test the performance of the models on the remaining data. This process is repeated for each student, and the mean and standard deviation of the results are reported across all students.

Figure 4 shows the F1 scores attained by different models. The first point (left most) on the plot represents the situation where there are no training data available for the left-out student. CALM-Net still outperforms Location MLP which is our baseline model. In this case, since there are no training data to train the personalized layers contained in CALM-Net and Branched CALM-Net, we will use the generic layer, which is trained with data from other students to make the predictions. Since branched CALM-Net has more trainable parameters, it would need more data points to achieve good performance, and it can be seen that with zero included data, CALM-Net and clustered CALM-Net outperform this model. When only 1 week of data is included from a student—meaning that the student has been using the application for at least 1 week—Branched CALM-Net outperforms other models.

We then inspect the performance of CALM-Net models on predicting binary stress labels (not stressed vs. stressed). The Branched CALM-Net achieves F1 scores of $0.883 \pm 0.023$ and $0.912 \pm 0.013$ with one and 2 weeks of data included, respectively, from left-out students. The findings we present not only demonstrate the effectiveness of our method when applied in a leave-one-subject-out setting but also align with our observations from the five-fold cross-validation experiments. Specifically, our results confirm that training the model with 3 levels of stress is adequately capable of handling the task of binary stress detection. This highlights the robustness and consistency of our approach.

### Results on WESAD dataset

To test the generalizability of our methodology, we apply the multi-heads personalization and dynamic branching methods to the Wearable Sensor and Affect Detection (WESAD)^51^ dataset.

The WESAD dataset is a freely accessible collection of multimodal data used for identifying stress and emotional states. This dataset includes physiological readings from 15 participants who were involved in three different activities: a baseline task involving 20 minutes of neutral reading, an amusement phase where they watched humorous videos for 392 seconds, and a stress-inducing segment using the Trier Social Stress Test for 10 minutes. During these activities, a variety of physiological parameters were recorded with devices worn on the chest and wrist. These parameters include blood volume pulse (BVP), electrocardiogram (ECG), electrodermal activity (EDA), electromyogram (EMG), respiration (RESP), body temperature, and accelerometer readings. The chest device recorded data at 700 Hz, while the wrist device did so at 64 Hz, 32 Hz, and 4 Hz. For data processing, we adopted the approach outlined in the study by Dzieżyc, Maciej, et al^52^, but we downsampled all signals to a uniform frequency of 4Hz to ensure consistency. For each participant, several personal attributes are used as covariates in the network to identify personalized differences. These attributes include age, height, weight, gender, handedness, whether they consumed coffee on the day of the experiment, their engagement in sports, smoking status, and whether they felt ill during the experiment.

Ultimately, the time series data is divided into one-minute segments, each labeled according to the specific task in which the participant was engaged during that minute. The objective is to predict the task being undertaken by the subject, using both their personal attributes and the data collected from the wearable sensors during that particular minute.

Table 6 shows the preliminary results under three-fold cross validation. Table 7 shows the leave-subject out validation with online learning setting. The empirical observation of models’ performance is consistent with the observation on the StudentLife^40^ dataset.

**Table 6:** The performance for 3 classes (baseline, stress, amusement) prediction on the WESAD dataset under three-fold cross validation.

Model	F1-score	ROC AUC
LSTM^46^ CALM-Net Branched CALM-Net	0.781 ± 0.023 0.794 ± 0.017 **0.805** ± **0.014**	0.883 ± 0.010 **0.938** ± **0.016** 0.929 ± 0.013
Transformer^46^ CATran-Net Branched CATran-Net	0.953 ± 0.008 **0.984** ± **0.012** 0.967 ± 0.030	0.995 ± 0.005 **0.999** ± **0.001** 0.988 ± 0.013

Empirical results from three-fold cross-validation, where the splits are stratified by subjects.

[bold] values indicate top performers in each category.

**Table 7:** The performance for 3 classes (baseline, stress, amusement) prediction on the WESAD dataset under leave-one-subject-out validation.

Model	F1-score $20\%$	F1-score $40\%$	F1-score $60\%$
LSTM CALM-Net Branched CALM-Net	0.758 ± 0.152 **0.772** ± **0.113** 0.753 ± 0.137	0.797 ± 0.140 **0.832** ± **0.087** 0.726 ± 0.159	0.784 ± 0.116 0.799 ± 0.116 **0.803** ± **0.109**
Transformer^46^ CATran-Net Branched CATran-Net	0.761 ± 0.145 **0.900** ± **0.100** 0.801 ± 0.166	0.791 ± 0.176 0.890 ± 0.118 **0.935** ± **0.093**	0.944 ± 0.091 0.938 ± 0.089 **0.960** ± **0.061**

The validation is conducted under leave-one-subject-out, with data from the left out subject being added to training set gradually to simulate the real-world online-learning setting. The percentage represent the amount of total available data included.

[bold] values indicate top performers in each category.

## Discussion

Early detection and intervention of stress are crucial for preventing the onset of various health problems. Smartphones, which are widely used, provide an opportunity for continuous monitoring of stress levels. However, the detection of stress from smartphone data poses several challenges. The data collected from smartphones are often irregular, noisy, and heterogeneous. *In our work, we show that by converting smartphone-derived physiological time-series data into a sequence of histograms and leveraging an auto-encoder, it is possible to substantially decrease the noise present in this data. This approach has demonstrated improved classification performance in forecasting student stress levels. *

This paper also proposes a novel multitask learning model with dynamic clustering called Branched CALM-Net, which aims to provide accurate stress prediction using data collected from smartphones. The proposed approach leverages the personalized structure to address intersubject variability. *By defining each student as a different task in a multitask neural network, the empirical results demonstrate notable enhancements in the stress prediction classification performance for the StudentLife dataset. This approach effectively captures the commonalities between students, while also adeptly modeling individual variations.*

The experimental results presented in “Results” section demonstrate that CALM-Net and Branched CALM-Net outperform the state-of-the-art methods^11,43^ in both binary diagnosis and stress level prediction tasks. This improvement can be attributed to the addition of personalized parameters that account for inter-subject variability. In addition, we observe that training the model with more fine-grained labels leads to better results. If we train CALM-Net directly on the binary stress labels, the precision in recall of $90\%$ is around $0.782 \pm 0.003$ with an AUC of $0.532 \pm 0.029$, worse than the performance shown in Table 2 and Fig. 3 where the models are trained with 3 distinct stress labels. This empirical evidence justifies our choice that *it is worth training with more fine-grained labels*, with a relatively balanced number of samples, and to merge the output probability when we need a coarse level of prediction.

The experiments conducted in the cold-start online learning setting, the results of which are summarized in Fig. 4, indicate that CALM-Net and Branched CALM-Net can quickly adapt to new subjects, *with Branched CALM-Net outperforming the other methods as long as the subject has at least 1 week of training data.*

Notably, Branched CALM-Net employs a learning-to-branch method that dynamically clusters students during the training process. This approach captures more valuable patterns from the data compared to using preclustered groups, as evidenced by the empirical results presented in “CALM-Net and Branched CALM-Net attain AUC scores of more than 0.8 on student stress detection” and “Branched CALM-Net improves upon the SOTA on 3-class stress level prediction” sections. Overall, Branched CALM-Net is able to cluster new students into a closer group, providing accurate stress level predictions after the first week of data collection as shown in Fig. 4. These findings demonstrate the potential of personalized multitask learning models with dynamic clustering for stress prediction using smartphone data.

*Following the solid empirical evidence of the performance of CALM-Net and Branched CALM-Net under both the warm-start and cold-start settings, we summarize our contributions as follows:*

(i) We proposed a novel platform for student stress prediction that models both population and personal characteristics with multitask learning and dynamic clustering. This model addresses the intersubject variability and cold-start problems.
(ii) We developed a data preprocessing pipeline that effectively reduces data noise and handles data irregularity to improve the accuracy of stress prediction.
(iii) We addressed an important but often ignored challenge in stress prediction by evaluating our models in the cold start setting. We added specific components to our model to handle this scenario, which enabled us to effectively predict stress levels even when we have little or no prior data for a given individual.

To sum up, our study demonstrates the ability of our system to effectively address the complex task of predicting student stress levels using mobile phone data. Our results suggest that it is possible to achieve automatic mental health monitoring through the use of deep neural networks. Our model’s accuracy is improved through the introduction of personalized multitask learning and dynamic clustering architecture, enabling it to provide accurate stress level predictions in various real-world scenarios with promising performance.

### Generalization and limitations

#### Scalability to large-scale populations and data privacy

While our model is designed with a unique neural network head for each student, facilitating personalization, this architectural choice inevitably leads to an increase in the model’s size in proportion to the number of participants. This presents a significant scalability challenge, especially since we have not yet had the opportunity to evaluate the model in a large-scale setting, due to the absence of appropriate datasets.

Notwithstanding this, the architecture of our model is inherently conducive to a federated learning approach. In such a large-scale application scenario, each student’s device would only maintain and update their individual neural network head. This modular design permits local updates and periodic synchronization with the overarching model framework, effectively mirroring a federated learning system^53–55^. Adopting this approach could provide a viable solution to the scalability issue by distributing the data processing and model training across devices. It is, however, crucial to note that both the implementation of this federated learning system and its validation in a large-scale context remain beyond the scope of our current research.

This proposed federated learning methodology also addresses key concerns regarding data privacy and security. The present model requires the sharing of student data with a server for periodic updates, which poses potential privacy risks. Yet, the modular structure of our model is ideally suited for a federated learning approach, wherein students would conduct updates on their individual network components locally. Consequently, only aggregated updates pertaining to the shared model components would need to be communicated to the server, significantly bolstering data privacy. Although the comprehensive implementation of this federated system is not covered in our existing research, it represents a promising avenue for future development, potentially ensuring scalability alongside enhanced privacy and security for practical applications.

#### Limitations and zero-shot performance

A notable limitation of our approach is its dependency on initial data for effective personalization. The model’s zero-shot performance, without prior data, is limited. This necessitates the collection of at least 1 week of labeled data from each student to achieve accurate stress predictions. To address this challenge, further integrating zero-shot learning techniques^56–58^ could potentially enhance the model’s initial performance for newcomers, thereby reducing the initial data dependency. Such advancements would be pivotal in making the model more robust and immediately useful for new participants.

#### Requirement of EMA stress labels and psychological surveys

While our models can perform effectively without psychological surveys, the EMA (Ecological Momentary Assessment) stress labels are crucial for training. These labels provide the necessary data for the models to learn and adapt to each student’s stress patterns. In cases where students cease to respond to EMAs, the model’s predictive accuracy may gradually decline due to the temporal shift in the student’s data. Addressing the challenge of declining student engagement with EMAs transcends the technical scope of our model and ventures into the realm of intervention strategies. It necessitates proactive outreach by school health staff to identify the causes behind the data gaps and evaluate the necessity for professional intervention. Furthermore, our framework holds the potential to be integrated with existing intervention tools, enhancing its practical utility. While the exploration of this integration is beyond the scope of the current paper, it represents a significant area for future research. Such studies could explore how our model can be symbiotically paired with intervention strategies to offer a more comprehensive solution for monitoring and addressing student stress.

*Adaptability to different demographics* Our current research primarily focuses on college students, a group with distinct stressors and challenges. However, the adaptability of our model to various demographics remains an area ripe for exploration. Different groups, such as high school students, students from diverse collegiate environments, or working professionals, encounter unique stressors that may not be fully encapsulated by our current model, which is tailored to the college student demographic. The performance and effectiveness of our model in these varied groups warrant thorough investigation. For instance, evaluating its applicability across different colleges, each with its unique demographic structure, is essential to understand the model’s versatility and effectiveness in diverse educational settings. Such evaluations are crucial, especially if the model is to be implemented practically in various institutions. Unfortunately, our current research was limited by the availability of datasets. We did not have access to extensive datasets encompassing these varied demographics, which restricted our ability to test and adapt the model across a broader demographic spectrum. Future research should aim to bridge this gap, focusing on gathering and analyzing data from these diverse groups. This approach will not only validate the model’s adaptability but also enhance its applicability and effectiveness in addressing the stress prediction needs of a wider population range.

## Methods

Since the survey data is taken from a publicly available dataset from a study conducted at Dartmouth College, no informed consent was required.

### Problem setup

We formulate the problem of predicting student stress levels as a supervised learning problem where the input to the model is the time series of the data collected from each student’s phone during the day and the label is the student stress level indicated by the student for that day. We denote the data collected from the student *i* from time step 1 to time step *T* as $X_{1:T}^{(i)} = \{x^{(i)}_1, x^{(i)}_2, \ldots , x^{(i)}_T\}$ and the stress label at time *T* as $y_T$. We want to predict $y_T$ given $X_{1:T}^{(i)}$.

We will illustrate our solution to this problem on the StudentLife dataset. In this dataset, students select a stress level from 1 to 5 each day. However, due to the uneven distribution of stress level labels, we have followed the approach proposed by^11^ and transformed the labels into a scale of 1 to 3. The new scale represents “below median stress”, “median stress”, and “above median stress”. Thus, the labels are now categorized as $y \in \{1, 2, 3\}$. The original stress levels of 1 and 2 have been mapped to “below median stress”, level 3 is “median stress”, and levels 4 and 5 have been mapped to “above median stress”. These three labels will be used for the supervised classification task.

### Baselines

We compared our models against the work by Gatis et al.^11^ titled MLP based on location features, which they called Location MLP. To extract features, Gatis et al.^11^ used GPS data and aggregated it on a daily basis. They extracted a total of eight location-based features, including the total distance covered, maximum displacement, distance entropy in 10-minute intervals, distance standard deviation, number of unique tiles visited, grid sections on the satellite map visited, the difference in tiles visited from the previous day, the approximate area of the GPS convex hull, and number of clusters in the GPS data. Additionally, they extracted four covariates based on the date, such as binary indicators of whether the day was the start of the term, the middle of the term, the end of the term, or a weekend.

We further evaluate our approach by contrasting it with the methodology outlined in^43^, which used a GBDT model for their predictions. In their study, they employed EMA data from the StudentLife dataset to extract a comprehensive set of 44 features. To mitigate class imbalance, they applied the SMOTE method. Their approach then determined the closest neighbors to a given sample using the Euclidean Distance metric, allowing for personalized modeling by training the model exclusively on the data of these nearest neighbors for each subject.

### Data pre-processing

**Table 8:** Feature preprocessing information.

Feature type	Feature name	Aggregation rule	Feature values	Imputation
Discrete	Activity	Mode	[0, 3]	Fill with zero
Sequences	Audio	Mode	[0, 3]	Fill with zero
	Conversation	Sum	[0, 1]	Forward fill
	Phone Charge	Sum	[0, 1]	Forward fill
	Phone Lock	Sum	[0, 1]	Forward fill, mean
	Time to deadline	N/A	[0, $\infty +$)	N/A
Covariates	Day of the week	N/A	[0, 6]	N/A
	Exam period	N/A	[0, 1]	N/A
	Sleep rating	Sum	[0, $\infty +$)	Forward fill, mean
	Sleep duration	Sum	[0, $\infty +$)	Forward fill, mean

During preprocessing, we first aggregate the time series data into bins of minutes, then compute the histogram within each hour. Here we list the aggregation rule, feature values, and modes in dataset for each feature. For the “mode” rule, we record whether events have happened. For the “sum” rule, we are extracting the number of times the events happened

**Figure 5:**
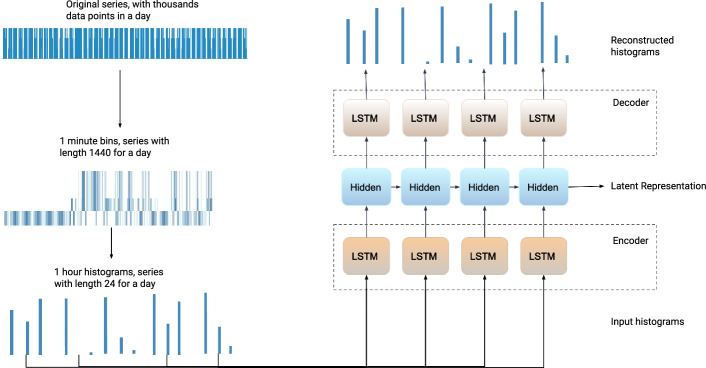
CALM-Net overall structure. We first aggregate each feature into 1-minute bins, then we compute the histogram for each hour. This data is then inputted into an LSTM Autoencoder for denoising and dimension reduction. The last hidden state is used as a latent representation of the entire input series, which is provided to the classification layers.

As passive sensors continuously collect data, there are thousands of measurements collected throughout the day for each student. Since these collections are noisy and it is difficult to model such a long time series data, we use feature engineering to reduce the noise and the length of this data. The signals are first binned with 1-minute blocks. Each feature is then binned with a different aggregation rule, as demonstrated in Table 8. Most of the features are aggregated by summation, representing the sum of the values of the feature over 1-minute bins, e.g., the total amount of conversation, phone locking time, sleep duration, etc. For *Activity* and *Audio* we use the mode to indicate whether the individual is performing an activity or if events are happening during that minute. So, by aggregating the data in this way, we remove small changes in short amounts of time, which is likely due to noise. The aggregated value of each bin forms the new series, with 1440 sequences per day. This series represents what the subject was doing at that minute. This sequence is still shown to be too long for recurrent neural networks^59^ so we further process the data by computing the histograms of 1-hour blocks. This step results in a sequence of 24 histograms per day. Figure 5 illustrates our feature engineering process. This conversion of the raw irregular time-series data to a series of histograms serves to reduce the noise and obtain regularly-sampled data.

Some of the features in the series exhibit missing values. Our imputation strategies for each feature are specified in Table 8. For most features, we adopt forward-fill imputation, where the missing time step is filled with that of the previous step. For the series with missing values at the beginning, we apply mean imputation after forward filling. For Activity and Audio, we use ‘0’ for the missing values and assume that nothing happened during those time steps.

This feature engineering technique actually models *‘how much conversation or activity a student has undergone in an hour which led to the stress label in a consistent manner’*. More specifically, our time series for a student will become a set of the following shape $X_{1:T}^{(i)}\in \mathbb {R}^{C \times T \times \hat{H}}$ as input to the model that contains the time series of hour histograms from one day. *C* is the number of channels such as activity, sound, and binary series; *T* is the length of the series, which is 24 in this case, and $\hat{H}$ is the size of the histogram.

Psychological surveys are also collected from students before the beginning and end of the study. The surveys include PHQ-9 for depression, PSS for stress, Loneliness scale, Flourish scale, PANAS, and the Big Five Personality Traits. Each survey could generate a score based on the student’s responses, representing the extent of the corresponding mental states, such as stress, loneliness, conscientiousness, etc. As a result, we have survey scores denoted as $S \in \mathbb {R}^{N \times \hat{S}}$ where *N* is the number of students and $\hat{S}$ is the number of surveys collected from students.

### Using auto-encoders to reduce data noise

Due to the nature of data collection from sensors on smartphones in the real world, there is a significant amount of noise present in the data. This noise can stem from factors such as the unreliability of the sensors, variations in phone placement, changes in environments, and more. While the data processing step mentioned earlier can remove some of this noise by aggregating the data into predefined bins, a substantial amount of noise will remain that could potentially harm the performance of models using this data. To address this issue,^41^ has summarized several practical techniques for modeling time series. We have chosen to use LSTM auto-encoders, as briefly illustrated in Fig. 5, to reduce this noise and obtain higher quality embeddings of the data.

The input to the LSTM encoder is a sequence of histograms as described in “Data pre-processing” section. Prior to being fed to the LSTM unit, the set of histograms is flattened at each time step. The output of the LSTM encoder is considered the latent representation of the input sequence and will be merged with covariates for subsequent classification processing. The decoder is employed to reconstruct the input sequence from the latent representation. The autoencoder is trained simultaneously with the entire model. We use the Mean Absolute Error (MAE) as the Reconstruction Error, denoted as *RE*. With the Classification Error, denoted as *CE*, we have the integrated loss:

1 $$\begin{aligned} {L_{integrated} = \alpha \cdot RE + \beta \cdot CE} \end{aligned}$$

where $\alpha$ and $\beta$ are the hyper-parameters ($0 \le \alpha , \beta \le 1$) for weighting the two losses. The choice of these coefficients depends on the relative importance of each loss. Given that the classification error is the primary task, we set its coefficient to 1. To prevent reconstruction loss, which serves as a denoising and regularization mechanism, from dominating the gradient during backpropagation, we set its coefficient to $1e-4$. We selected these values through hyperparameter optimization.

Both our LSTM encoder and decoder consist of a single LSTM layer. The last hidden state of the encoder with a size of 128 is used as the latent representation of the input data. The decoder will take the hidden states from the encoder at all time steps as input and predicts the original sequence.

### Personalization

**Figure 6:**
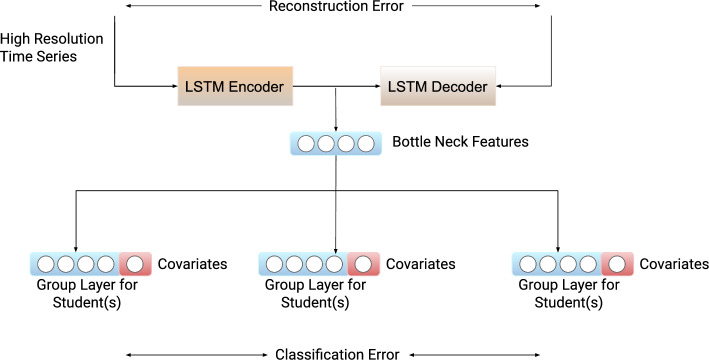
The structure of Multitask Learner, an LSTM autoencoder followed by multi-heads output. All the group layers have their own parameters, and they share the LSTM autoencoder along with the bottle-neck features. It is equivalent to CALM-NET if each group layer contains exactly one student.

It is crucial to consider individual differences when modeling mental states such as stress, as these experiences can vary greatly between individuals^27^. This attribute should be taken into account when modeling data from mobile phones for the purpose of stress prediction. Busk et al.^17^ and Jaques et al.^16^ have emphasized the importance of personalized parameters when modeling individual moods. Kandemir et al.^60^ approach the personalization in the prediction of affect (mood) by learning user-specific kernels, further strengthening the significance of modeling individual patterns. To learn personalized models for each student, we adopt a multitask approach that consists of an LSTM to model the sequence of histograms, followed by shared fully connected layers and a MLP for each student.

Specifically, our model has a 2-layer MLP for each student on top of a shared fully connected layer and the LSTM Auto-encoder. Each fully connected layer has a ReLU activation function except the last layer, which maps the input to the class probabilities. We named this proposed model the Cross-Personal Activity LSTM Multitask Auto-Encoder Network (CALM-NET). Figure 6 represents a CALM-Net model where each group layer contains exactly one student—thus becoming a personalization layer.

As indicated by our experiments in “Results” section, this approach can learn the differences between students and subsequently yield a significant improvement in performance. Furthermore, according to our ablation studies presented in the Appendix Section C, learning a single model for all the students is unsuited to this task. Multitask learning also acts as a heavy regularizer, preventing the model from overfitting to a single student or the most common label. The shared layers learn common features, while the personalization layers learn features that are relevant to the respective subject.

A drawback of this approach is that we need training data for every student. If a new student joins the system, the model will not be able to make predictions for the new student. This is called the “cold-start problem” in the literature. To solve this problem, we developed and tested different approaches, as outlined in the following subsections.

#### Clustering students

The first approach we used to address the cold start problem was to cluster students into groups that have similar characteristics. We then predict the stress level for a group of students instead of individuals. In this way, for a newly added student, we first assign them to a group and then use the predictive model corresponding to the group to obtain the individual prediction.

We use the student surveys to group them. In this way, students with similar characteristics who answered to surveys similarly will be clustered together. Then, we replace the personalized layers in the model architecture with group layers, where we use an MLP for each group of students.

Specifically, the features used for grouping are aggregated scores from the surveys, which are collected before the beginning of the study: Big Five, Flourishing Scale, Loneliness Scale, Positive and Negative Affect Schedule, Perceived Stress Scale, and PHQ-9. The clustering method that we applied is Density-Based Spatial Clustering of Applications with Noise (DBSCAN)^61^. The hyper-parameters were tuned during experiments. The empirical results show that models with 3 to 4 students in each cluster on average achieve the best possible performance. The model has the structure shown in Fig. 6, where the students are assigned to each group layer based on the survey scores.

#### Branched CALM-NET

The clustering approach relies on static clustering using data collected during surveys at the beginning of data collection. However, this approach may be limited by the availability of data, and the inability of the survey data collected at the beginning of the study to capture changes in students’ behaviors over time. To overcome this limitation, we developed a model that dynamically clusters students into groups while predicting their stress levels. Our approach is based on the “learning to branch” method originally proposed by^34^. Our model includes a shared layer followed by group nodes, with individual layers for each student connected to all group nodes. By training on the data, the model learns branches in the network that assign each individual to the best group, enabling clustering and classification. A diagram of the model architecture is provided in Fig. 1.

More formally, each personalized layer will receive the output from different group nodes with a likelihood distribution. An illustration of the pipeline is shown in Fig. 2. We will denote the output of the group node *i* as $B_i$, the likelihood of the personalized layer *j* receiving the output from the branch *i* as $\theta _{i, j}$. Each personalized layer will take the output from the branch with the largest likelihood. Then the output, denoted as $d_j$, which will be received by the personalized layer *j* is

$$\begin{aligned} d_j = B^T one\_hot \{argmax_i(\theta _{i, j})\} \end{aligned}$$

However, as $\frac{\partial d_j}{\partial \theta _{j}}$ is not directly differentiable, it will not work with the backpropagation algorithm in training the neural network. In order to force the probability close to the one-hot vector during training and still make the branching operation differentiable, we replace the one-hot operation with *Softmax*. We used *the Gumbel trick*, as stated in^62^, which is adopted as a solution provided in^34^. By applying the Gumbel softmax trick, a smooth version of the branching operation is given as:

$$\begin{aligned} d_j = B^T \frac{\exp ((\log \theta _{j} + \epsilon ) / \tau )}{\sum _k \exp ((\log \theta _{k, j} + \epsilon _k) / \tau )} \end{aligned}$$

The noise term $\epsilon$ is generated from the Gumbel distribution, as described by^62^, to prevent the network from being overly sensitive to parameter initialization. This is crucial because if the network were to favor branches with higher likelihood at initialization, it could result in branches with lower likelihood suffering from gradient saturation during training. The temperature parameter $\tau$ controls the smoothness of softmax operation, with smaller values making the output closer to a one-hot vector. During training, we initialize $\tau$ with a relatively large value, then linearly decay it across epochs.

Therefore, at the end of the training, subjects that choose the same branch will be considered as being in the same group.

### CATrans-Net and branched CATrans-Net

Similar to CALM-Net and Branched CALM-Net we introduce CATrans-Net and Branched CATrans-Net which have similar structures to CALM-Net and Branched CALM-Net but replace the LSTM autoencoder with the Transformer model introduced by Foumani et al. ^46^ in the model architecture.

### Adding the generic layer

Since the CALM-Net and Learn-to-Branch models have personalized parameters, they cannot make predictions for a new incoming student without any prior training data available. To address this, we added a generic layer to both models, as shown in Fig. 1, which is trained using data from all students in the training set. By incorporating a multitask approach, the integrated loss function becomes:

$$\begin{aligned} L_{integrated} = \alpha \cdot RE + \beta \cdot CE_{personal\_layer} + \lambda \cdot CE_{generic\_layer} \end{aligned}$$

The integrated loss function includes three components: *RE*, which is the reconstruction loss from the auto-encoder part of the model; $CE_{personal\_layer}$, which represents the classification loss from individual student layers; and $CE_{generic\_layer}$, which denotes the classification loss from the generic layer. To balance the optimization rate and account for the fact that the parameters of the generic layer are optimized in every iteration, we set the weight assigned to the classification loss with output from the generic layer ($\lambda$) to one over the number of students (excluding the new incoming student), which is 22 in this particular dataset.

## Conclusion

In this study, we introduced CALM-Net and its branched version, Branched CALM-Net, for stress level prediction in the StudentLife dataset. We used feature engineering and histogram categorical inference to address noise and align different signals in the data. We also employed LSTM-Autoencoder to further reduce data noise and overfitting, as well as to improve the quality of the embeddings. We implemented a Multi-Task network structure with personalized parameters for each subject to address inter-subject variability. We also enhanced our model through the Learn-to-Branch to identify similar groups of students and thus improve prediction performance.

Both CALM-Net and Branched CALM-Net achieved peak performance with an *F1-score* of 0.602 and 0.605, respectively, in the five-fold cross-validation setting in the 3-level stress classification task. Branched CALM-Net outperformed all other models with just 1 week of training data available under leave-one-subject-out. In the binary diagnosis task, Branched CALM-Net maintained a *precision* of over 84% and 82%, while *recovering* 90% and 95% of positive cases, respectively. These results suggest that the characteristics at both group-level and individual-level are significant in modeling the pattern of passive sensor data from various subjects.

In the future, we plan to explore our model’s capabilities for forecasting tasks, where instead of only predicting the current stress level, the model is trained to forecast future stress levels. Additionally, we aim to use this model for the prediction of other mental well-being indicators such as mood. Ultimately, our platform is meant to assist as a guide for early intervention.

### Supplementary Information

Supplementary Information.

## Data Availability

The datasets generated during and/or analysed during the current study are available at: https://studentlife.cs.dartmouth.edu/dataset.html.

## Code availability

The source code used for conducting experiments and generating results during the current study are available in a public repository on GitHub: https://github.com/Information-Fusion-Lab-Umass/personalized-stress-prediction.
