# Supplementary material for: Dynamic clustering via branched deep learning enhances personalization of stress prediction from mobile sensor data
Source: Sci Rep. 2024 Mar 19;14:6631. doi: 10.1038/s41598-024-56674-2 (PMC10951234; doi:10.1038/s41598-024-56674-2)
Supplement: Supplementary file 1 — Supplementary Information. [file 41598_2024_56674_MOESM1_ESM.pdf]

Appendix A Used Features

Our data comprises of 23 students, totaling 1183 data points achieving roughly equal amount of training data in [11]. These 1183 data points have the following label distribution - 263 below median stress, 511 median stress, and 409 above median. The rest of the students that have the number of labels much higher than the average are removed from the training set as they may dominate the shared modules of the models.

The list of student IDs used for training are - [4, 7, 8, 10, 14, 16, 17, 19, 22, 23, 24, 32, 33, 35, 36, 43, 44, 49, 51, 52, 53, 57, 58]

The input to the model are both feature engineered time series and the covariates. The details of these input features are shown in Table 4.

Appendix B Model Configurations

The model configuration for Branched CALM-Net and hyper-parameters are given in Table B1

**Hyper-parameter**	**value**
α	1e-4
β	1
λ	$\frac{1}{22}$
Auto-encoder embedding size	128
Branching layer hidden size	256
Personal layer hidden size	64
Learning Rate	1e-5
L2 Penalty	1e-4

Table B1: The configuration details of Branched CALM-Net. The Learn-to-Branch layers will replace the shared layers. And each branch has a hidden size equal to the replaced shared layers.

Appendix C Ablation Study

In Tables C3 and C2, we present the results of our ablation study. The term "LSTM Autoencoder" denotes the addition of a decoder to the LSTM model, while still utilizing the bottleneck as input for the linear classification layer. We also experimented with CALM-Net without covariates and a pre-trained autoencoder, whereas our final version of CALM-Net incorporates covariates by concatenating them and simultaneously training the autoencoder alongside the primary task. For the Branched CALM-Net, the presented results employ vanilla Softmax for branching selection, whereas our ultimate version employs the Gumbel-Trick Softmax.

Table C2: More Empirical Results on Performance Evaluation in Binary Stress Detection

Model	Precision (@Recall≈0.9)	Precision (@Recall≈0.95)	AUC
LSTM Autoencoder	0.782 ± 0.002	0.783 ± 0.002	0.528 ± 0.007
CALM-Net No covariates	0.842 ± 0.003	**0.823±0.002**	**0.807 ± 0.002**
CALM-Net Pre-trained AE	**0.847 ± 0.004**	0.821 ± 0.003	0.803 ± 0.003
Branched CALM-Net (with Softmax)	0.843 ± 0.003	0.818 ± 0.003	0.806 ± 0.002
Location MLP	0.588 ± 0.002	0.588 ± 0.002	0.580 ± 0.011
LSTM	0.780 ± 0.004	0.781 ± 0.003	0.530 ± 0.020
CALM-Net	0.843 ± 0.003	**0.818 ± 0.003**	**0.807 ± 0.002**
Branched CALM-Net	**0.845±0.005**	0.816±0.002	0.805±0.004

The labels of *median stress* and *very stressed* are combined, then evaluate the precision while fixing recall at 90% and 95%.

Table C3: More Empirical Results on performance of stress level prediction on StudentLife dataset under 5-fold Cross Validation

Model	F1-score	AUC
LSTM Autoencoder	0.478±0.004	0.631±0.002
CALM-Net No covariates	0.586±0.003	0.767±0.002
CALM-Net Pre-trained AE	0.601±0.005	**0.780±0.001**
Branched CALM-Net (with Softmax)	0.601±0.003	0.778±0.003
Location MLP	0.388±0.007	0.631±0.003
LSTM	0.479±0.004	0.630±0.002
**CALM-Net**	0.602±0.004	0.778±0.002
**Branched CALM-Net**	**0.605±0.003**	**0.782±0.002**

Performances on the task of stress level prediction with 3 levels (below median, median, above median). The reference performance are shown below the split line.
